# Dealing with an aging China—Delaying retirement or the second-child policy?

**DOI:** 10.1371/journal.pone.0242252

**Published:** 2021-01-07

**Authors:** Yantao Ling, Zhe Song, Yang Yu, Tangyang Jiang

**Affiliations:** 1 School of Economics and Business Administration, Chongqing University, Chongqing, China; 2 Department of Economics, Pusan National University, Busan, Republic of Korea; 3 School of Internet, Anhui University, Anhui, China; Institute for Advanced Sustainability Studies, GERMANY

## Abstract

To tackle China’s rapidly aging population, a policy was framed by using overlapping generations (OLG) model and computable general equilibrium (CGE) model; the main objective was to successfully implement “second-child policy” and “delayed retirement age” for female or male workers. The 2012 census data was obtained from National Bureau of Statistics of China. Our research findings suggest that the economy can be improved in the short-term by delaying retirement age; however, Chinese economy would improve tremendously in the long run by implementing second-child policy. Compared to delayed retirement age, second-child policy would be more effective in improving the economy in China. In terms of industrial output, the three policies have a greater influence on labor-intensive industries, such as agriculture, light industry, finance, and service sector; the impact is less significant on construction and heavy industry. In terms of industrial import and export, these three policies have greatly influenced following industries: finance, electric power, and fossil energy. From a monetary perspective, these three policies can significantly improve household income; these three policies did not significantly impact both government and corporate incomes.

## Introduction

**Fig 1:**
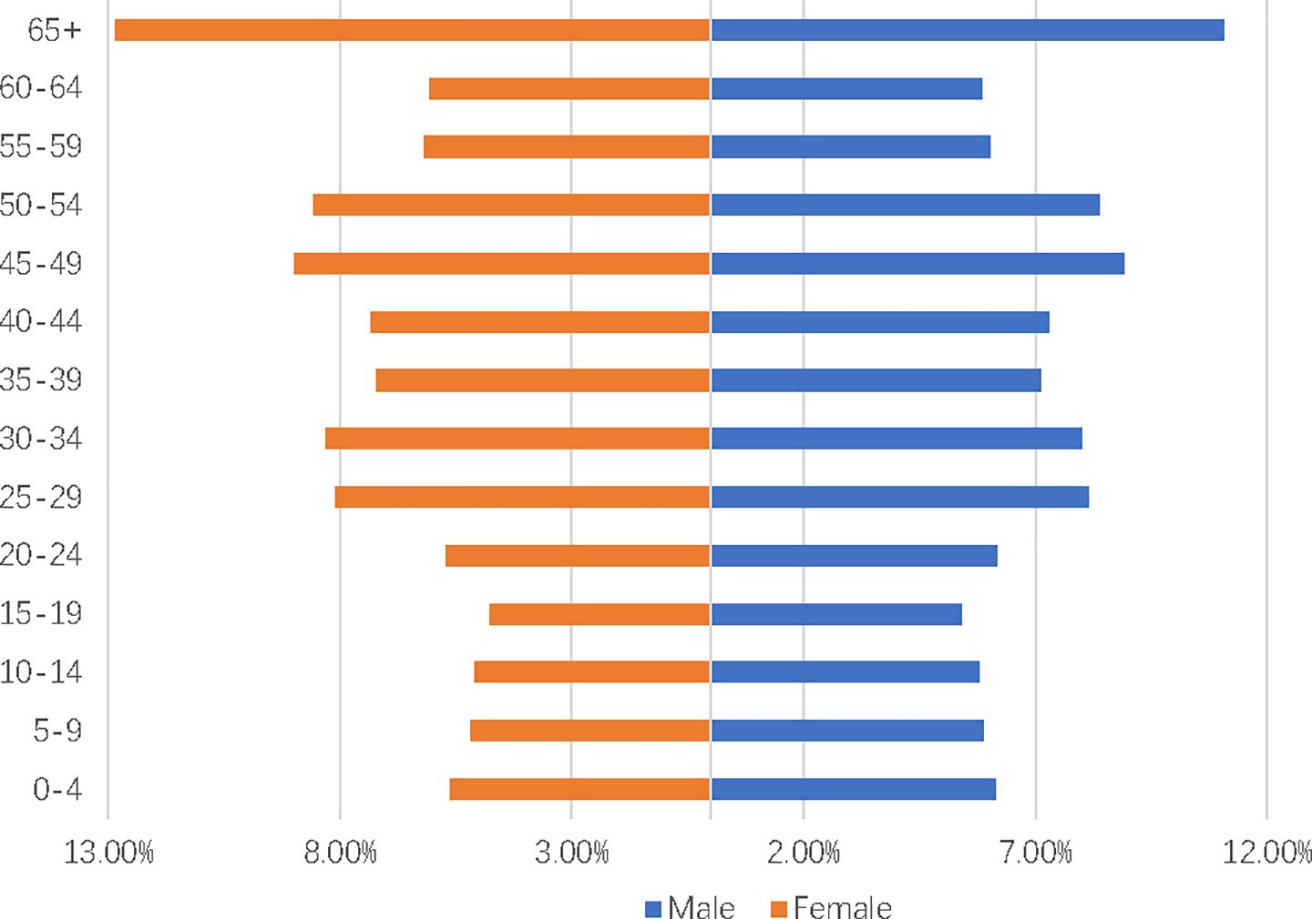
China’s population pyramid of 2018. The data is retrieved from Chapter 3–10 Population by Age and Sex in the 2019 China Statistical Yearbook. We aggregated the data for 65 years old and above into one group while the remaining data is kept unchanged.

The one-child policy, a part of family planning policy, became the basic national policy of China in 1982. The Chinese government’s main intention was to control population explosion; however, one-child policy was only enforced on Han Chinese group, which constitutes 92% of the Chinese population. One-child policy was not enforced on the remaining ethnic minorities; there are 55 ethnic minorities in China till date. It has been almost 40 years since the Chinese government implemented one-child policy; however, the problem of population explosion has now turned into a problem of increasing aging population. This is because life expectancy of Chinese citizens has increased substantially, while birth rate has declined tremendously in China. The birth rate has dwindled from 2.10% in 1990 to 1.29% in 2016, whereas the elderly dependency ratio (the ratio of people older than 64 to those aged between 15 and 64) has increased from 8.30% to 15.00%. Fig 1 illustrates the distribution of Chinese population across various age groups: females are presented on the left side of the coordinating axis, while males are presented on the right side of the coordinating axis. The population is segregated into 5-year age groups, with the youngest age group being at the top and the oldest age group being at the bottom. In this pyramid, the population of Chinese citizens aged above 65 years is exceedingly greater than those of other age groups; the population was extremely low for the age groups ranging from 0 to 19; this could be attributed to the declining birth rate in China. Fig 1 summarizes the current demographic situation in China. If the current trend continues, the birth rate would decline sharply in China. Meanwhile, the elderly dependency ratio would increase tremendously. Currently, policy makers and economists are trying hard to develop innovative solutions that can cope with China’s aging society. For example, the Chinese government introduced two-child policy recently to tackle the declining birth rate. Moreover, the official retirement age may be increased by the Chinese government.

Compared to the aging problem of other countries, China’s aging issue holds its own special characteristics for following two reasons: (1) China’s birth rate has declined steadily ever since the implementation of one-child Policy in 1980s; moreover, a large difference is observed in the population of different age groups. Chinese families have always favored sons over daughters [1], leading to a severe imbalance between men and women of the same age group [2]. (2) Chinese government implemented second-child policy in 2016; however, we still do not know whether this policy can effectively solve China’s aging problem. Therefore, Chinese government is most likely to adopt “delaying retirement policy”. This policy would certainly increase labor force, and it would also alleviate accompanying pressures on public finances. In this study, we tried to address following questions: How would these policies work at the same time? What is the impact of these policies on Chinese economy? Would there be any conflicts in implementing these policies? To address these questions, we compared these policies comprehensively; the analyses results were used to critically examine these policies.

At this stage, the main intention of policy makers is to solve the aging issue in China. Several scholars have conducted extensive research studies to understand different perspectives of aging issue Many scholars think that aging population has increased tremendously due to two reasons: (i) a declining birth rate and (ii) an increasing life expectancy [3–5]. The same two factors have led to China’s aging issue [6]. The aging population has caused three types of negative impacts: (i) increased financial burden of government, (ii) a low saving rate, and (iii) a slowdown in economy [7]. Because of the steep increase in aging population, Chinese government has to incur a large expenditure of pensions. Moreover, the cost of eldercare has also increased tremendously, which falls on the government for an increased social assistance payment. In totality, aging population has become a major financial burden on for regional governments [8–11]. In another research study, it was reported that an aging society would lead to a decrease in household saving rate [12–14]. Ando and Modigliani (1954) [15] proposed the Life Cycle Theory of Consumption: retirees can sell off their assets to provide for food, housing, and recreation in retirement homes; this would contribute to a dissaving status. Nathaniel (1969) [16] used cross-country data to justify this hypothesis: he concluded that there is a negative relationship between elderly dependency ratio and national savings rate. This phenomenon has also been confirmed in subsequent studies [17–20]. The economy is negatively impacted by an aging society [21–23]. Most studies have reported that more social resources would be consumed by a society, which has an excessive proportion of older population. This would inhibit reproduction, increasing social burden and disrupting a sustainable economy. For example, a severe aging problem disrupts the balance of economic development. Owing to changes in age structure, an older society would lead to disappearance of demographic dividend and household debt problems. A comprehensive study was conducted to determine economic issues in Australia; an aging population is considered to be one of the most dominating factors to hinder economic development in coming decades [24]. Most scholars have presented many theories for solving negative effects of aging. To tackle aging issue, most scholars have recommended following two approaches: (i) the official retirement age must be delayed and (ii) the fertility rate must be improved [25–27]. The Institute of Medicine (US) Committee on the Long-Run Macroeconomic Effects of the Aging U.S. Population (2012) [28] conducted a large-scale study to determine the long-term macroeconomic effects of aging population in the report “Aging and the Macroeconomy: Long-Term Implications of an Older Population”; they pointed out that the short-term solution would be to delay retirement age; the long-term remedy would be to improve fertility rate. Thus, the population structure can be optimized effectively.

There were only few studies on the second-child policy as an antidote to an aging society. Most studies assume that retirement age and fertility rate are endogenous; this implies that individuals can choose their optimal retirement age in most countries [29]. In China, retirement age and fertility rate are mostly determined exogenously by the government. The legal retirement age is 60 and 50 for male and female workers, respectively; however, the retirement age is 55 for female public servants. The second-child policy was recently introduced by the Chinese government. Thus, the fertility rate of Chinese couples is usually governed exogenously by the Chinese government (Most couples in China are willing to have more than one child.).

Based on shortcomings of the existing literature on the “second-child policy” and “delaying retirement policy”, we considered these “three policies” as exogenous factors in our model, where Policies 1 and 2 investigate the economic impact of delayed retirement and Policy 3 analyzes the impact of second-child policy. Specifically speaking, Policy 1: Holding all else equals, only female labors are delayed by five years; Policy 2: Holding all else equals, only male labors are delayed by five years; Policy 3: Holding all else equals, only the birth rate is doubled. Then, we analyzed their effects on China’s economy from a demographic and gender perspective. Ours is the first study to investigate how China is grappling with its aging problems by the second child policy. On a macro level, we analyzed effects on gross domestic product (GDP). On a micro level, we analyzed effects on various sectors in terms of outputs, imports, exports, and income. Finally, we drafted policy recommendations and considerations to provide theoretical basis and reference for future studies.

Our study has three theoretical contributions. (1) We construct a research framework that can quantitatively analyze the economic effects of population policies by combining OLG and CGE models with labor rate as an intermediate variable. (2) Our study expands the research perspective to assess the economic effects of delayed retirement policy by distinguishing the gender differences. In assessing the economic effects of the delay retirement policy, we considered the gender differences and evaluated the economic impact of male delayed retirement and female delayed retirement, respectively. (3) This study further completes the assessment of the short-term and long-term economic impact of the two-child policy and the delayed retirement policy, and then comparing the similarities and differences of the two policies.

The rest of the paper is organized as follows: Section 2 describes policy design. Section 3 presents modeling framework. The results of numerical simulations are presented in Section 4. A conclusion and its implications are presented in Section 5.

## Policy design

The constitution of People’s Republic of China states that the official retirement age for men and women is 60 and 50 years, respectively. In the public sector, female workers can retire at 55 years. If the national economy shows a noticeable decline, the Chinese government can delay the “official retirement age” and adopt “second-child policy.” This would help the country to cope with its economic decline through legislation.

We simulated three demographic policies: Policy Ⅰ (a five-year delay for female workers), Policy Ⅱ (a five-year delay for male workers), and Policy Ⅲ (second-child policy). In simulation scenario, the 2012 birth rate, mortality, and net growth rate were used as baseline. The detailed policy design is presented in Table 1.

**Table 1:** Policy design for delayed retirement and second child policy.

Policies	Policy Description
Baseline Situation	With the birth rate and mortality rate in 2012 as the birth rate and mortality rate for each year after 2012 to 2037. Male labor force is defined as working males in the age group of 15–59 years. Female labor force is defined as working females in the age group of 15–49 years. The sum of the two is the total labor force.
Policy Ⅰ	With the birth rate and mortality rate in 2012 as the base birth rate and base mortality rate for each year after 2012 to 2037. Male labor force is defined as working males in the age group of 15–59 years. Female labor force is defined as working females in the age group of 15–54 years (a five-year delay). The sum of the two is defined as the total labor force.
Policy Ⅱ	With the birth rate and mortality rate in 2012 as the base birth rate and base mortality rate for each year after 2012 to 2037. Male labor force is defined as working males in the age group of 15–64 years (a five-year delay). Female labor force is defined as working females in the age group of 15–49 years. The sum of the two is defined as the total labor force.
Policy Ⅲ	With the mortality rate and doubled birth rate for 2012 as the base mortality rate and base birth rate for each year after 2012 to 2037. Male labor force is defined as working males in the age group of 15–59 years. Female labor force is defined as working females in the age group of 15–49 years. The sum of the two is defined as the total labor force.

The Sixth National Population Census of China was published in 2010. The average life expectancy for males and females is 72.38 years and 77.37 years, respectively. The delayed retirement policy has its own statistical reasoning. Table 2 presents the data for age and gender structure in China.

**Table 2:** The age and gender structure in China.

Age	0–4	5–9	10–14	15–19	20–24	25–29	30–34	35–39	40–44	45–49	50–54	55–59	60–64	65+
Male	3.08	2.96	2.88	3.46	4.63	4.02	3.78	4.05	4.88	4.5	2.81	3.21	2.48	4.51
Female	2.6	2.49	2.44	3.11	4.42	3.97	3.65	3.87	4.68	4.33	2.7	3.14	2.45	4.9

**Note:** The data was retrieved from Chapter 3–10 Population by Age and Sex in the 2013 China Statistical Yearbook. We aggregated the data for citizens aged 65 years and above; this data was presented into one group. The remaining data was kept unchanged.

After acquiring the data, we need to estimate China’s future population by age and sex. To achieve this objective, we referred to several scholarly articles. We decided to adopt the Overlapping Generation Model, which was presented by Paul Samuelson in 1958. This economic model was considered as the most classical and mainstreamed “representative agent.” The model assumes that agents can live for a finite length of time. The life of each agent can be divided into three phases: schooling, working, and retirement. Therefore, agents move from one period to another period throughout their life overlapping with other agents’ life.

Bernanke and Gertler (1989) [30] used an overlapping generations (OLG) model to improve the actual business economic cycle; the results were published in American Economic Review. A financial accelerator model was constructed to determine the causes of business cycle fluctuations in terms of the financial market. Auerbach and Kotlikof (1989) [31] first created an intergenerational model to simulate the effect of pension reform and aging population on capital accumulation. They used this model to determine how changes in population age structure impacted the following four OECD (Organization for Economic Cooperation and Development) countries: the United States of America, Japan, Germany, and Sweden. They also used this model to simulate the impact of privatization on the social security system of USA. Persson and Tabellini (1994) [32] developed another OLG model to determine the effect of income distribution on economic growth and wealth redistribution. The inequality they study is reflected in their personal ability or endowment. Higher the ability or endowment, higher is the income.

An OLG model was used to process the data in a different manner. The population data was acquired and aggregated into a five-year group; we could not divide various phases of life into three categories: schooling, working, and retirement. We had to create more phases of life. We could not focus on individual economic activities that occurred within each phase of life. Instead, we emphasized the total amount of labor produced from each phase of life. Based on the above two factors, we constructed an OLG model with 14 phases of life; this model does not focus on the economic activities of an individual within each phase of life.

**Fig 2:**
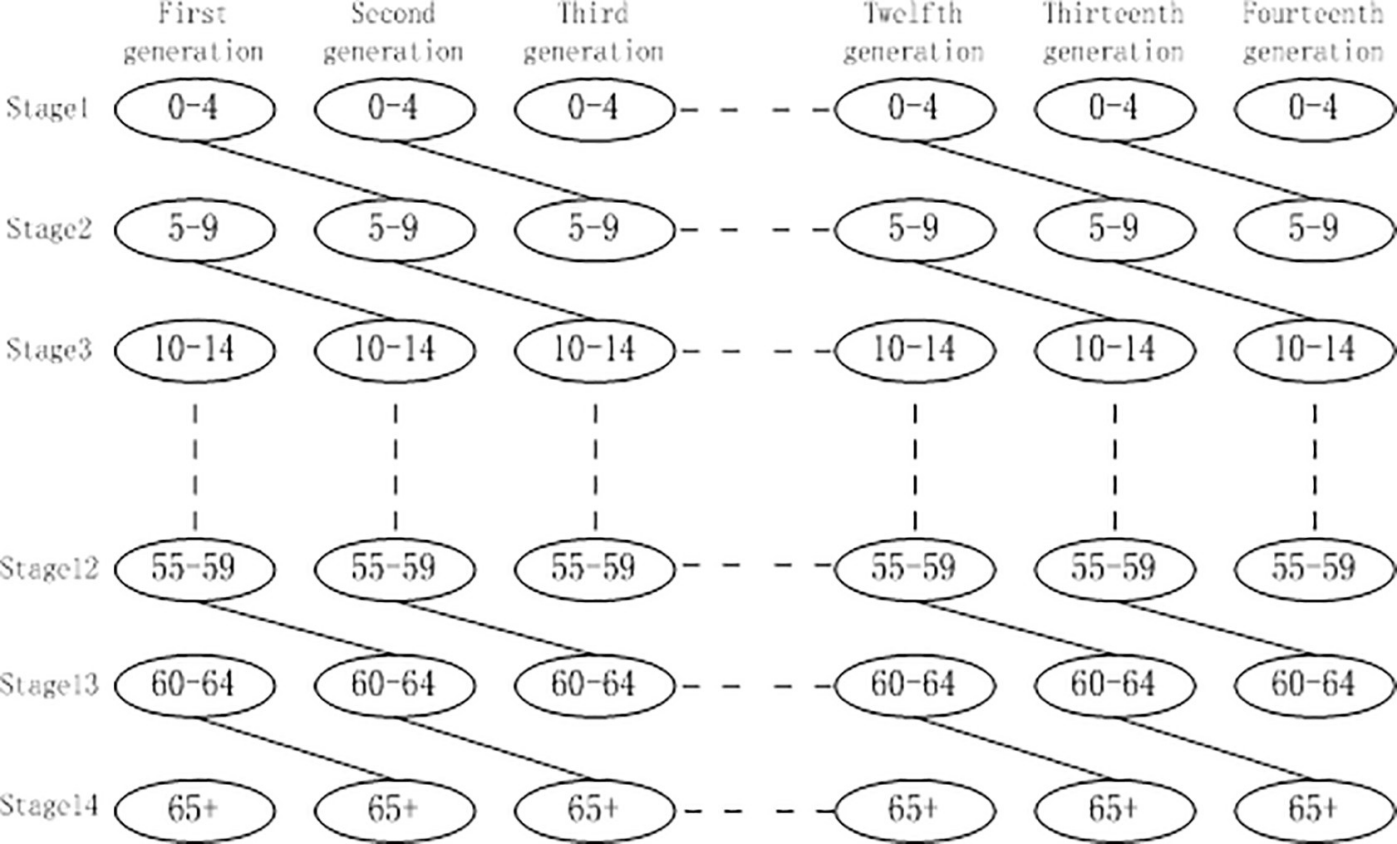
Population structure map.

Using the data mentioned above, we divided the population into 14 groups; each group consisted of people within five-year age bracket. The population structure was updated every five years: newborns were included into the age group of 0–4 years; toddlers were segregated into the age group of 5–9 years. The generation map was illustrated in Fig 2.

The population structure data was updated every five years. The annual population data was obtained from the National Bureau of Statistics in China; we processed this data to fit into our model. We started population analyses from the year 2012. Let “a” denote the birth rate every year. Let “b” denote the rate of natural increase in population. The share of each age group of males was denoted as *c*_*i*_ (i = 1, 2, 3…14); the share of each age group of females was denoted as *d*_*i*_(i = 1, 2, 3…14). Moreover, “Y” represents the birth rate every five years and “X” denotes the rate of natural increase in birth rate every five years. We have,

$$Y=\sum_{j=0}^{4}a{\left(1+b\right)}^{j}$$ (1)

$$X=\sum_{j=0}^{4}{b(1+b)}^{j}$$ (2)

This means that Y represents the population share of group 1 (age 0–4) after the first five years and will be the population share of group 2 (age 5–9) after the second five years. The population share of males in group 1 (age 0–4) after i (i = 1,2,…5) five-years is *W*_*i*_ (i = 1,2…5). The population share of females in group 1 (age 0–4) after i five-years (i = 1,2,…5) is *U*_*i*_(i = 1,2…5). Then we have,

$$W_{i}=\frac{Y}{2}*{\left(1+X\right)}^{i-1}$$ (3)

$$U_{i}=\frac{Y}{2}*{\left(1+X\right)}^{i-1}$$ (4)

Let *Z*_*i*_ denote the population share of males aged between 15 to 59 years after i five-years from 2012. Let *K*_*i*_ be the population share of females aged between 15 to 49 years after i five-years from 2012. The total labor share is L.

$$Z_{i}=\sum_{j=4}^{12-i}c_{j}+\sum W_{i}$$ (5)

$$K_{i}=\sum_{j=4}^{10-i}d_{j}+\sum U_{i}$$ (6)

$$L=Z_{i}+K_{i}$$ (7)

Using the data and model mentioned above, we estimated the proportions of male aged between 15 to 59 years and female populations aged between 15 to 49 years under the baseline situation. Using Policy I, we also estimated the proportions of male population aged between 15 to 59 years; moreover, we estimated the proportions of female population aged 15 to 54 years. Using Policy II, we estimated the proportions of male population aged between 15 to 64 years; moreover, we also estimated the proportions of female population aged between 15 to 49 years. Under Policy III, we estimated the proportions of male population aged between 15 to 59 years; we also estimated the female population aged between 15 to 49 years. Table 3 presents the labor supply under different scenarios.

**Table 3:** Labor supply under different scenarios.

Policies	2012	2017	2022	2027	2032	2037
Baseline Situation	1	0.965	0.933	0.89	0.852	0.826
Policy Ⅰ	1.039	1.016	0.977	0.961	0.929	0.89
Policy Ⅱ	1.043	1.033	1.007	0.951	0.91	0.889
Policy Ⅲ	1	0.965	0.933	0.89	0.951	1.054

**Fig 3:**
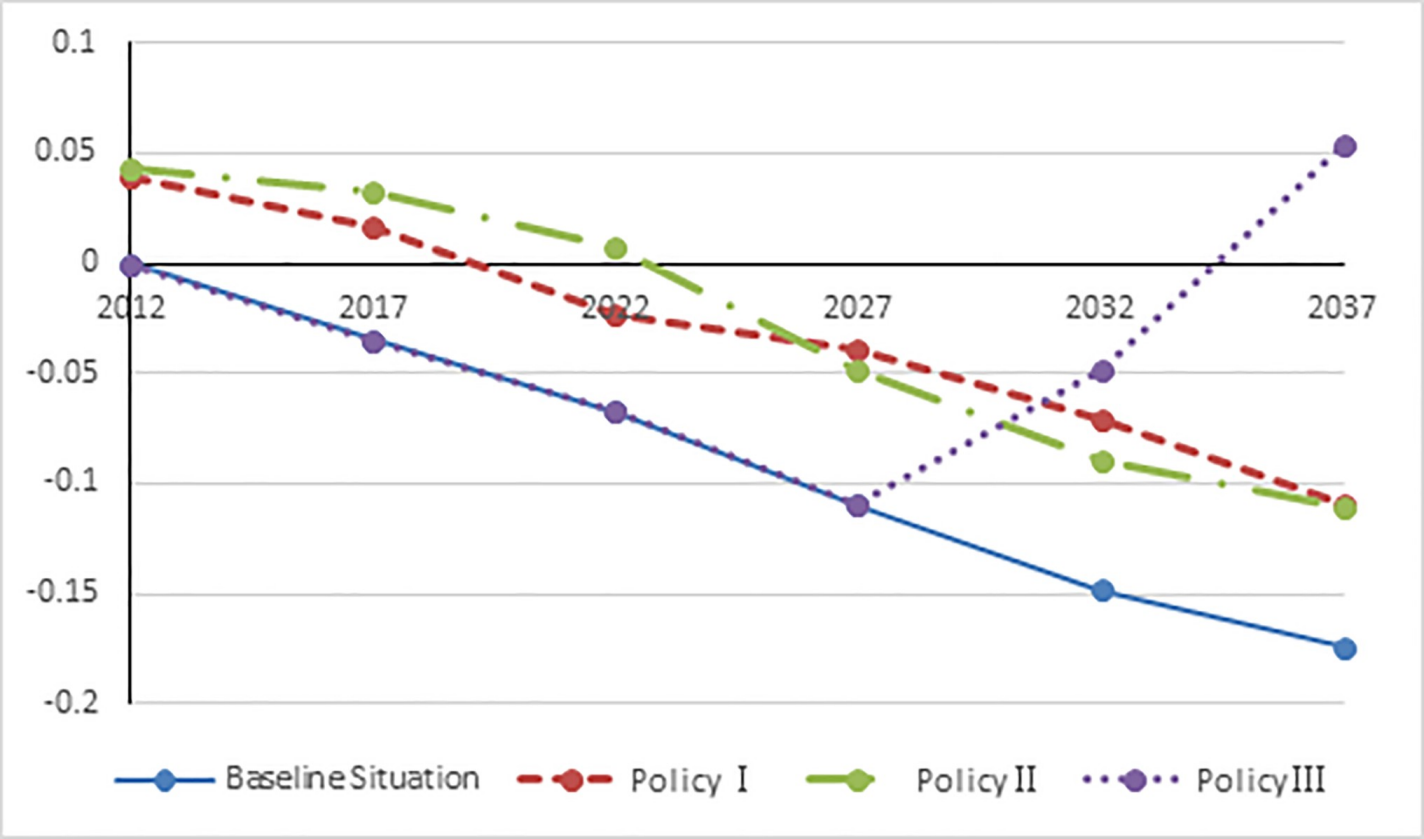
The influence of labor under three demographic policies.

The data shown in Table 3 proves that China’s aging problem is escalating without any population policy. When we implement Policy Ⅰ or Policy Ⅱ, short-term labor shortage caused by China’s aging population is alleviated; however, it cannot compensate for long-term labor shortage. By implementing Policy Ⅲ, labor can be increased significantly in the long term; however, short-term labor shortage cannot be tackled by implementing Policy III. The influence of labor is as follows shown in Fig 3.

## Data and model

In this paper, based on the static CGE model, a population dynamics module is added. The biggest contribution of this paper is that in the population dynamics module, the population increase parameter is not the net population growth rate in the previous researches, but is the working age population. The CGE model and data is described as follows [33, 34].

### Data

In this study, data was sourced from the 2012 extended input-output table of 42 producing sectors; these sectors were defined by the National Bureau of Statistics of China. As shown in Table 4, we aggregated the data and land on the input-output table of 10 producing sectors [35]. To compile SAM, we obtained data from various databases. The data on activities, commodities, and international trade were obtained from the 2012 input-output table. The data on government income and spending, tax revenue, foreign subsidies, quantity of traded goods and services, and household income and expenditure were retrieved from following books: the 2013 Fiscal Yearbook of China, the 2013 China Taxation Yearbook, the 2013 China Customs Statistical Yearbook, and the 2013 China Statistical Yearbook.

**Table 4:** The sector classification.

Sectors	Content
1 Agriculture	Agriculture, Forestry, Animal Husbandry, and Fishery
2 Fossil Energy	Coal Mining and Processing, Oil Mining and Processing, Natural Gas Mining and Processing, Production and Supply of Gas
3 Electric Power	Production and Supply of Electric Power and Heat Power
4 Heavy Industry	Metal Ores Mining and Processing, Non-metal Ores Mining and Processing, Other Mining Products, Non-metallic mineral products, Metal Smelting, Steel Rolling Processing, Metal Products, General Equipment Manufacturing, Special Equipment Manufacturing, Transportation Equipment Manufacturing, Electrical Machinery and Equipment Manufacturing, Telecommunications Equipment Manufacturing, Electronic Computer Manufacturing
5 Light Industry	Foods and Alcoholic Beverages, Tobacco Products, Textile Materials Processing, Textile, Knitting Products Manufacturing, Textile, Clothing, Shoes, Hats Manufacturing, Leather, Fur, and Feathers and Its Products, Wood Processing and Furniture Manufacturing, Paper Making, Printing, Stationary and Sporting Goods Manufacturing, Instrument Manufacturing, Other Manufacturing Products, Waste Materials, Production and Supply of Water
6 Chemical Industry	Chemical Products Manufacturing
7 Construction	Construction
8 Transportation	Transportation and Warehousing, Postal Service
9 Finance	Finance
10 The Service Industry	Metal Products, Comprehensive Technical Service, Post and Telecommunications Information, Computer Services and Software Wholesale and Retail Trade, Accommodation and Catering Industry, Real Estate, Leasing and Business services, Research and Experimental Development Industry, Water Conservancy, Environment and Public Facilities Management, Residents Service and Other Services, Education, Health and Social Security, Culture, Sports and Entertainment, Public Administration and Social Organization

There were variations in the quality of data provided by yearbooks; some accounts have been unavoidably not balanced. Therefore, the minimum cross-entropy method was used to balance accounts. To balance SAM and to solve CGE model, we employed the Non-linear Programming Planning and the Mixed Complementarily Programming respectively. Finally, the macro and micro SAM was obtained for the open economy in China [36–40].

### Model

Let us construct a CGE model for China under an open economy; the factors of production are labor and capital. We also aggregated 42 producing sectors into 10, and we regarded their inputs as intermediate inputs. For simplicity, we presented a few core equations [41–44].

1) Production Equation

The relationship between input and output was described by the production function. We used Constant Elasticity of Substitution (CES) function for added value and intermediate inputs. The equation is as follows.

$${QA}_{i}={a_{i}^{q}[\delta_{i}^{q}QVA_{i}^{\rho_{i}}+(1-\delta_{i}^{q}){QINTA}_{i}^{\rho_{i}}]}^{\frac{1}{\rho_{i}}}$$ (8)

where, QA_i_ is total output of sector i, QVA_i_ is total added value (labor and capital) of sector i, QINTA_i_ is intermediate input of sector i, $a_{i}^{q}$ is shift parameter of sector i, $\delta_{i}^{q}$ is share parameter of sector i, ρ_i_ is exponent in the CES function.

2) Trade-Related Equations

We assume that China is a small economy, so its domestic market price is not enough to affect the world price. Therefore, there are no perfect domestic substitutes for imported goods and services. We used Armington function: the quantity imported was represented by CES production function, and the quantity exported was represented by Constant Elasticity of Transformation (CET) function. The equations are as follows:

$${QA}_{i}=\alpha_{i}^{t}{\left[\delta_{i}^{t}{QDA}_{i}^{\rho_{i}^{t}}+(1-\delta_{i}^{t}){QE}_{i}^{\rho_{i}^{t}}\right]}^{\frac{1}{\rho_{i}^{t}}}$$ (9)

where, QA_i_ is total output of sector i, QDA is total domestic consumption of sector i, QE_i_ is total export of sector i.

$${QQ}_{C}=\alpha_{c}^{q}{\left(\delta_{c}^{q}{QDC}_{c}^{\rho_{c}^{q}}+(1-\delta_{c}^{q}){QM}_{c}^{\rho_{c}^{q}}\right)}^{\frac{1}{\rho_{c}^{q}}}$$ (10)

where, QQ_C_ is quantity supplied of good c in domestic market, QDC_c_ is quantity consumed of good c in domestic market, QM_c_ is quantity imported of good c.

3) Price-Related Equations

Three equations are involved in price-related equations:

$$\frac{{PVA}_{i}}{{PINTA}_{i}}=\frac{\delta_{i}^{q}}{\left(1-\delta_{i}^{q}\right)}{\left(\frac{{QINTA}_{i}}{{QVA}_{i}}\right)}^{1-\rho_{i}}$$ (11)

where, PVA_i_ is price of added value in sector i, PINTA_i_ is price of intermediate inputs in sector i, QVA_i_ is quantity of added value in sector i, QINTA_i_ is quantity of intermediate input in sector i, $\delta_{i}^{q}$ is share parameter in sector i.

$$\frac{{PDA}_{i}}{{PE}_{i}}=\frac{\delta_{i}^{t}}{\left(1-\delta_{i}^{t}\right)}{\left(\frac{{QE}_{i}}{{QDA}_{i}}\right)}^{1-\rho_{i}^{t}}$$ (12)

where, PDA_i_ is price of sector i in the domestic market, PE_i_ is price of sector i in the foreign market, QDA_i_ is quantity consumed of sector i in the domestic market, QE_i_ is quantity exported of sector i.

$$\frac{{PDC}_{c}}{{PM}_{c}}=\frac{\delta_{c}^{q}}{\left(1-\delta_{c}^{q}\right)}{\left(\frac{{QM}_{c}}{{QDC}_{c}}\right)}^{1-\rho_{c}^{q}}$$ (13)

where, PDC_c_ is price of good c in the domestic market, PM_c_ is price of good c from the foreign, QDC_c_ is quantity consumed of good c in the domestic market, QM_c_ is quantity imported of good c from the foreign market.

4) Income-Related Equations

There are mainly three types of income in a CGE model: household income, corporate income, and government income. Household income is obtained from salaries, wages, transfer payments, and subsidies. Corporate income is obtained from return on investment. Government income is obtained from taxes and tariffs, which are collected from different subjects in the economy. The three equations are as follows.

$$YH=WL*QLS+WK*{shif}_{hk}*QKS+{transfr}_{hent}+{transfr}_{hgov}+{transfr}_{hrow}$$ (14)

where, YH is total household income, WL is price of labor, QLS is supply of labor, WK is price of capital, QKS is supply of capital, shif_hk_ is share parameter of household income to capital income, transfer_hent_ is transfer payments from firms to households, transfer_hgov_ is transfer payments from government to households, transfer_hrow_ is transfer payments from foreign enterprises to households.

$$YENT=WK*QKS*{shif}_{entk}$$ (15)

where, YENT is total corporate income, QKS is total supply of capital, shif_entk_ is share parameter of corporate income to capital income.

$$YG=tva(WL*QLD+WK+QKD)+{ti}_{h}*YH+{ti}_{ent}*YENT+\sum_{c}{tm}_{c}*{pwm}_{c}*{QM}_{C}*EXR$$ (16)

where, YH is total household income, YENT is total corporate income, WL is price of labor, QLD is total quantity demanded of labor, WK is price of capital, QKD is total demand of capital, tva is gross production tax, ti_h_ is personal income tax, ti_ent_ is corporate income tax, pwm is prices of commodities after tariffs in foreign currency, tm_c_ is tariffs on imported good c, QM_C_ is quantity imported of good c, EXR is exchange rate.

5) Expenditure-related Equations

There are three equations involved in expenditure-related equations: household consumption, corporate expenditure, and government spending. The three equations are as follows:

$${PQ}_{c}*{QH}_{c}={shrh}_{c}*mpc*(1-{ti}_{h})*YH$$ (17)

where, PQ_c_ is price of good c in household consumption, QH_c_ is quantity consumed of good c in household consumption, shrh_c_ is marginal propensity to consume of good c, mpc is marginal propensity to consume of all goods and services, ti_h_ is personal income tax, YH is total household income.

$$EG=\sum_{i}{PQ}_{i}*{QG}_{i}+{transfr}_{hg}+GSAV+{transfr}_{rowg}$$ (18)

where, PQ_i_ price of good i in government spending, QG_i_ is quantity consumed of good i in government spending, transfr_hg_ is government transfer payment to households, transfr_rowg_ is government subsidies to foreign countries, GSAV is government saving.

$$ENTSAV=(1-{ti}_{ent})YENT-{transfr}_{hent}$$ (19)

where, ENTSAV is corporate saving, ti_ent_ is corporate income tax rate, YENT is total corporate revenue, transfr_hent_ is transfer payments to households.

6) Population Growth Dynamic Equation

$$\lambda_{t+n}^{tfp}=(1+Ratepg)\lambda_{t}^{tfp}$$ (20)

where, $\lambda_{t+n}^{tfp}$ is labor supply after n years based on $\lambda_{t}^{tfp}$, *Ratepg* is population growth rate.

7) Market Clearance and Macro Closure

A macro closure is reflected by three main equations that represent following components: (i) the balance between saving and investment, (ii) the balance of government financial payments, and (iii) the balance of trade. When equilibrium is attained in a perfectly competitive market, it implies that the market is clear; this indicates that equilibrium exists between following parameters: price mechanism, factor market, commodity market, and current account.

The CGE model can be solved by three methods: General Algebraic Modeling System (GAMS), Mathematical Programming System for General Equilibrium (MPSGE), and General Equilibrium Modeling Package (GEMPACK). In general, GAMS is the most common one among the three methods; it adopts an algebraic definition to approximate the standard. The mathematical expression of a CGE model can be easily converted into a GAMS programming language, which is suitable for complex mathematical modeling on a large scale. Besides, linear, non-linear, and mixed-integer optimization problems can be specifically solved with GAMS programming language. Therefore, we used GAMS programming language to solve CGE model.

## Stimulation results and analysis

In this study, we used CGE model to simulate three demographic policies (Policy I represent delayed retirement age for female workers. Policy II represents delayed retirement for male workers. Policy III represents second-child policy). Then, we analyzed the economic impact of these policies on China’s future economy, industrial output, industrial imports and exports, household income, corporate income, and government income. The detailed simulation results are as follows.

### The impact of GDP

**Fig 4:**
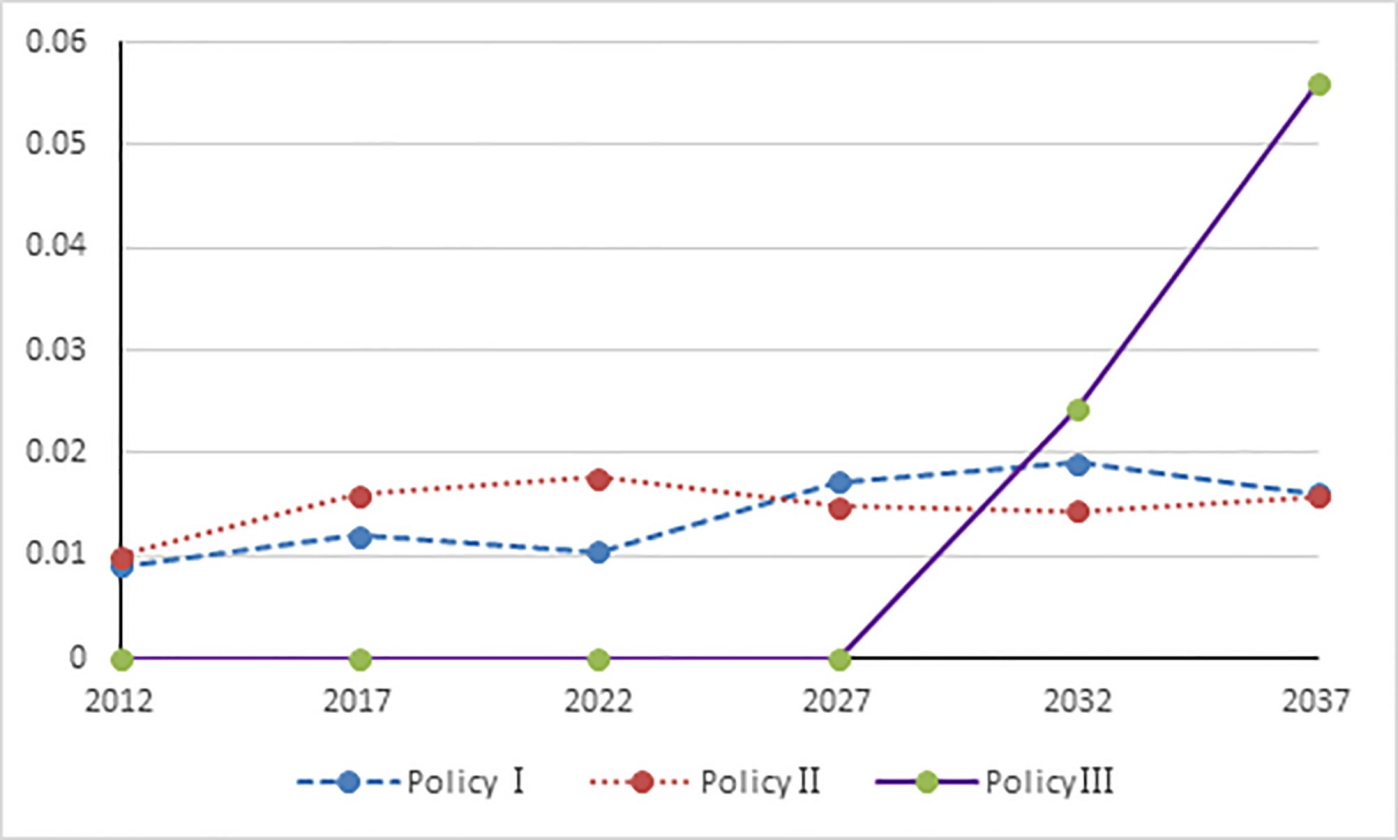
Real GDP under three policies.

Fig 4 illustrates that compared to the baseline scenario, the delayed retirement policies have a stable and sustainable driving effect on the economy, and the two-child policy plays a strong driving force long-term economic growth. More specifically, the three policies of the economy differ in terms of degree and time efficacy. Starting from 2012, delayed retirement for male workers has a stronger impact on GDP than that for female workers. This trend would continue until 2027 when the delayed retirement age of female workers would have a stronger impact on GDP. From 2012 to 2027, second-child policy would have a slight economic impact on GDP. However, second-child policy would have a better economic impact from the year 2027.

The analyses results indicate that compared to second-child policy, delayed retirement age has a stronger economic impact in the short term. In the long term, second-child policy has a stronger economic impact than delayed retirement age. Delayed retirement for male and delayed retirement for female have different economic effects, and the two policies have cyclical effects on the economy This is because labor force could be increased by directly delaying the retirement age of male and female workers. The increased labor force was directly related to production process as a factor of production, which immediately increases the GDP. Although birth rate would increase with two-child policy, newborns cannot be added to the labor market; therefore, industrial production would not increase in the short term due to two-child policy. This implies that second-child policy cannot instantly increase the GDP of production sector. However, second-child policy would certainly increase the number of newborns in due course of time. After a certain period of time, these newborns would become adults and participate in production as they are added to active labor force; this would promote domestic production and improve China’s GDP. In terms of age and gender, the demographic structure is not balanced in China; the ratio of men and women is not completely equal across all age groups. In some age groups, the proportion of males is greater than that of females. In another age group, the proportion of females is greater than that of males. The same gender is different. The proportion of age group is not exactly the same. In fact, the difference is large. Therefore, it leads to the same number of years for men and women to postpone their retirement. This has led to differences in the strength of economy; the economy showed a cyclical phenomenon of drop and rise due to the strengths and weaknesses of policies.

To successfully tackle China’s aging issue, the Chinese government should implement “second-child policy”. To alleviate the financial burden of having a second child and increase the enthusiasm of families to have a second child, we suggest the Chinese government to provide birth incentives to citizens. In the short term, the economic impact of second-child policy is not quite significant. But, in the long term, labor shortage can be alleviated by effectively implementing second child policy. This would also help in optimizing the population structure of China. If there is a considerable decline in China’s economy in the short term, male and female workers may be delayed at the same time and gradually increase their retirement ages. We do not recommend a uniform delayed retirement policy for male or female workers, because the single-delayed retirement policy has a more volatile impact on the economy. It also avoids giving the economy too little or too much stimulus at times.

#### The impact of industrial output

Compared to baseline scenario, delaying retirement and the second-child policy have positive impact on the economy (Table 5). During the same time period, the same policy has a different economic impact on the output of different sectors. In the first five years of policy implementation, Policy I would increase the output of agriculture, light industry, finance, and services sector by 16.1‰, 13.5‰, 10.3‰, and 9.9‰, respectively. The output of construction and heavy industry would increase by 0.3‰ and 3.8‰. Policy II increases the output of agriculture, light industry, finance, and service sector by 17.7‰, 14.9‰, 11.3‰, and 10.9‰, respectively. The output of construction and heavy industry would increase by 0.3‰ and 4.2‰. Policy III reveals slight economic impact on these sectors.

**Table 5:** Industrial output by sector.

Policy	Sector	2012	2017	2022	2027	2032	2037
Policy Ⅰ	Agriculture	0.0161	0.0214	0.0189	0.0312	0.0346	0.0293
	Fossil Energy	0.0090	0.0120	0.0105	0.0172	0.0190	0.0160
	Electric Power	0.0085	0.0112	0.0099	0.0162	0.0179	0.0151
	Heavy Industry	0.0038	0.0051	0.0044	0.0072	0.0079	0.0067
	Light Industry	0.0135	0.0180	0.0159	0.0262	0.0291	0.0246
	Chemical Industry	0.0096	0.0127	0.0111	0.0183	0.0202	0.0171
	Construction	0.0003	0.0004	0.0003	0.0006	0.0006	0.0005
	Transportation	0.0088	0.0117	0.0103	0.0169	0.0187	0.0158
	Finance	0.0103	0.0137	0.0120	0.0198	0.0219	0.0185
	Service	0.0099	0.0131	0.0115	0.0189	0.0209	0.0176
Policy Ⅱ	Agriculture	0.0177	0.0286	0.0317	0.0268	0.0261	0.0288
	Fossil Energy	0.0099	0.0159	0.0176	0.0148	0.0144	0.0158
	Electric Power	0.0093	0.0150	0.0165	0.0139	0.0135	0.0148
	Heavy Industry	0.0042	0.0067	0.0074	0.0062	0.0060	0.0065
	Light Industry	0.0149	0.0240	0.0266	0.0226	0.0220	0.0243
	Chemical Industry	0.0105	0.0169	0.0187	0.0157	0.0153	0.0168
	Construction	0.0003	0.0005	0.0006	0.0005	0.0005	0.0005
	Transportation	0.0098	0.0156	0.0173	0.0146	0.0141	0.0155
	Finance	0.0113	0.0182	0.0201	0.0170	0.0165	0.0182
	Service	0.0109	0.0174	0.0193	0.0163	0.0158	0.0174
Policy Ⅲ	Agriculture	0.0000	0.0000	0.0000	0.0000	0.0444	0.1028
	Fossil Energy	0.0000	0.0000	0.0000	0.0000	0.0244	0.0560
	Electric Power	0.0000	0.0000	0.0000	0.0000	0.0229	0.0526
	Heavy Industry	0.0000	0.0000	0.0000	0.0000	0.0101	0.0232
	Light Industry	0.0000	0.0000	0.0000	0.0000	0.0373	0.0860
	Chemical Industry	0.0000	0.0000	0.0000	0.0000	0.0259	0.0596
	Construction	0.0000	0.0000	0.0000	0.0000	0.0008	0.0018
	Transportation	0.0000	0.0000	0.0000	0.0000	0.0240	0.0550
	Finance	0.0000	0.0000	0.0000	0.0000	0.0280	0.0644
	Service	0.0000	0.0000	0.0000	0.0000	0.0268	0.0616

Policy III exhibits a noticeable economic influence after the year 2032. And, the economic impact of Policy III on industrial output was stronger than that of Policy I and Policy II. Policy III increases the economic output of agriculture, light industry, finance, and service sector by 44.4‰, 37.3‰, 28.0‰ and 26.8‰ respectively. The output of construction and heavy industry is increased by 0.8‰ and 10.1‰, respectively. Policy I increases the output of agriculture, light industry, finance, and service sector by 34.6‰, 29.1‰, 21.9‰ and 20.9‰, respectively. It also increases the output of construction and heavy industry by 0.6‰ and 7.9‰, respectively. Policy II increases the output of agriculture, light industry, finance, service sector, construction and heavy industry by 26.1‰, 22.0‰, 16.5‰, 15.8‰, 0.5‰ and 6.0‰, respectively.

Compared to the baseline scenario, the three demographic policies significantly impact agriculture, light industry, finance, and service sector; however, they produce a smaller impact on construction and heavy industry. This is because agriculture, light industry, finance, and service sector are labor intensive industries. By implementing these three policies, more labor can be introduced into corresponding industrial sectors. The outputs would be enhanced consequently. All policies are conducive to increasing the share of agriculture and the third industry and promoting the optimization of China’s industrial structure. To ensure these policies can promote industrial structure upgrading and achieve the desired results, it is necessary that these industries adopt appropriate policy measures.

#### The impact of industrial import and export

Compared to baseline scenario, the three demographic policies have positive economic impact on industrial imports except for the construction industry (Table 6). In addition, these demographic policies also have a positive economic impact on industrial exports; however, agriculture and construction industry are an exception. Within five years of implementing Policy I, the imports in finance, electric power, transportation, fossil fuel, agriculture, and heavy industry increased by 11.1‰, 9.2‰, 7.7‰, 7.5‰, 0.6‰, and 1.7‰, respectively. Moreover, the exports of finance, electric power, construction and fossil fuel, heavy industry and agriculture also increased by 11.4‰, 9.5‰, –6.9‰, 6.7‰, 0.4‰ and –2.2‰, respectively. Following the implementation of Policy II, the imports of finance, electric power, transportation, fossil fuel, agriculture and heavy industry increased by 12.2‰, 10.1‰, 8.5‰, 8.2‰, 0.6‰ and 1.8‰, respectively. The imports of finance, electric power, construction, fossil fuel, heavy industry and agriculture increased by 12.6‰, 10.5‰, –7.6‰, 7.4‰, 0.5‰ and –2.4‰, respectively. However, Policy III showed little economic impacts on these sectors.

**Table 6:** Industrial import and export by sector.

Policy	M&X	Sector	2012	2017	2022	2027	2032	2037
Policy Ⅰ	Import	Agriculture	0.0006	0.0008	0.0007	0.0013	0.0015	0.0013
		Fossil Energy	0.0075	0.0099	0.0087	0.0142	0.0157	0.0133
		Electric Power	0.0092	0.0122	0.0107	0.0176	0.0195	0.0164
		Heavy Industry	0.0017	0.0022	0.0019	0.0031	0.0034	0.0028
		Light Industry	0.0070	0.0093	0.0082	0.0135	0.0150	0.0127
		Chemical Industry	0.0076	0.0101	0.0089	0.0146	0.0161	0.0136
		Construction	-0.0046	-0.0061	-0.0054	-0.0088	-0.0098	-0.0083
		Transportation	0.0077	0.0102	0.0090	0.0147	0.0163	0.0137
		Finance	0.0111	0.0147	0.0129	0.0213	0.0236	0.0199
		Service	0.0074	0.0099	0.0087	0.0142	0.0157	0.0133
Policy Ⅰ	Export	Agriculture	-0.0022	-0.0029	-0.0025	-0.0041	-0.0044	-0.0037
		Fossil Energy	0.0067	0.0089	0.0078	0.0129	0.0142	0.0120
		Electric Power	0.0096	0.0127	0.0111	0.0183	0.0202	0.0170
		Heavy Industry	0.0004	0.0005	0.0004	0.0007	0.0007	0.0006
		Light Industry	0.0043	0.0057	0.0050	0.0083	0.0092	0.0078
		Chemical Industry	0.0070	0.0092	0.0081	0.0133	0.0146	0.0123
		Construction	-0.0069	-0.0091	-0.0080	-0.0131	-0.0145	-0.0123
		Transportation	0.0063	0.0084	0.0073	0.0121	0.0133	0.0113
		Finance	0.0114	0.0152	0.0133	0.0220	0.0243	0.0206
		Service	0.0062	0.0083	0.0073	0.0120	0.0132	0.0112
Policy Ⅱ	Import	Agriculture	0.0006	0.0011	0.0012	0.0011	0.0011	0.0013
		Fossil Energy	0.0082	0.0132	0.0145	0.0123	0.0119	0.0131
		Electric Power	0.0101	0.0163	0.0180	0.0151	0.0147	0.0161
		Heavy Industry	0.0018	0.0029	0.0032	0.0027	0.0025	0.0028
		Light Industry	0.0077	0.0123	0.0137	0.0116	0.0113	0.0125
		Chemical Industry	0.0084	0.0135	0.0149	0.0126	0.0122	0.0134
		Construction	-0.0051	-0.0082	-0.0090	-0.0076	-0.0074	-0.0081
		Transportation	0.0085	0.0136	0.0150	0.0127	0.0123	0.0135
		Finance	0.0122	0.0196	0.0217	0.0183	0.0178	0.0196
		Service	0.0082	0.0131	0.0145	0.0122	0.0119	0.0131
Policy Ⅱ	Export	Agriculture	-0.0024	-0.0038	-0.0042	-0.0035	-0.0033	-0.0036
		Fossil Energy	0.0074	0.0119	0.0131	0.0111	0.0107	0.0118
		Electric Power	0.0105	0.0169	0.0186	0.0157	0.0152	0.0167
		Heavy Industry	0.0005	0.0007	0.0008	0.0006	0.0005	0.0006
		Light Industry	0.0047	0.0076	0.0084	0.0072	0.0070	0.0077
		Chemical Industry	0.0077	0.0123	0.0135	0.0114	0.0111	0.0122
		Construction	-0.0076	-0.0121	-0.0134	-0.0113	-0.0110	-0.0121
		Transportation	0.0069	0.0111	0.0123	0.0104	0.0101	0.0111
		Finance	0.0126	0.0202	0.0224	0.0189	0.0184	0.0202
		Service	0.0069	0.0110	0.0122	0.0103	0.0100	0.0110
Policy Ⅲ	Import	Agriculture	0.0000	0.0000	0.0000	0.0000	0.0018	0.0040
		Fossil Energy	0.0000	0.0000	0.0000	0.0000	0.0202	0.0462
		Electric Power	0.0000	0.0000	0.0000	0.0000	0.0250	0.0573
		Heavy Industry	0.0000	0.0000	0.0000	0.0000	0.0043	0.0099
		Light Industry	0.0000	0.0000	0.0000	0.0000	0.0192	0.0436
		Chemical Industry	0.0000	0.0000	0.0000	0.0000	0.0206	0.0473
		Construction	0.0000	0.0000	0.0000	0.0000	-0.0125	-0.0279
		Transportation	0.0000	0.0000	0.0000	0.0000	0.0209	0.0478
		Finance	0.0000	0.0000	0.0000	0.0000	0.0302	0.0695
		Service	0.0000	0.0000	0.0000	0.0000	0.0202	0.0461
Policy Ⅲ	Export	Agriculture	0.0000	0.0000	0.0000	0.0000	-0.0057	-0.0129
		Fossil Energy	0.0000	0.0000	0.0000	0.0000	0.0182	0.0416
		Electric Power	0.0000	0.0000	0.0000	0.0000	0.0259	0.0595
		Heavy Industry	0.0000	0.0000	0.0000	0.0000	0.0009	0.0022
		Light Industry	0.0000	0.0000	0.0000	0.0000	0.0118	0.0267
		Chemical Industry	0.0000	0.0000	0.0000	0.0000	0.0188	0.0430
		Construction	0.0000	0.0000	0.0000	0.0000	-0.0185	-0.0412
		Transportation	0.0000	0.0000	0.0000	0.0000	0.0171	0.0390
		Finance	0.0000	0.0000	0.0000	0.0000	0.0312	0.0718
		Service	0.0000	0.0000	0.0000	0.0000	0.0169	0.0387

Policy III exerted its economic growth effect only in the year 2032, this implies that the second-child policy can have a big impact on the economy in the long run. Meanwhile, the economic impact of Policy III on imports and exports was stronger than that of Policy I and Policy II on imports and exports in the same period. Policy III increased the imports of finance, electric power, transportation, fossil fuel, agriculture and heavy industry by 30.2‰, 25.0‰, 20.9‰, 20.2‰, 1.8‰ and 4.3‰, respectively. In terms of exports, Policy III also increased the exports of finance, electric power, construction, fossil fuel, heavy industry and agriculture by 31.2‰, 25.9‰, –18.5‰, 18.2‰, 0.9‰ and –5.7‰, respectively. Policy I increases the imports of finance, electric power, transportation, fossil fuel, agriculture and heavy industry by 23.6‰, 19.5‰, 16.3‰, 15.7‰, 1.5‰ and 3.4‰, respectively. Policy I also increases the exports of finance, electric power, construction, fossil fuel, heavy industry and agriculture by 24.3‰, 20.2‰, –14.5‰, 14.2‰, 0.7‰ and –4.4‰, respectively. Policy II increases the imports of finance, electric power, transportation, fossil fuel, agriculture and heavy industry by 17.8‰, 14.7‰, 12.3‰, 11.9‰ 1.1‰ and 2.5‰, respectively. Policy II also increases the exports of finance, electric power, construction, fossil fuel, heavy industry and agriculture by 18.4‰, 15.2‰, –11.0‰, 10.7‰, 0.5‰ and –3.3‰, respectively.

The analyses results are as follows: compared to the baseline scenario, the same policy has the same degree of impact on the same sector at the same time. Moreover, the impact of the three demographic policies on imports was stronger than that of export; however, agriculture, electric power, construction and finance industries were exceptions. This is because the demand for production activities would increase following the implementation of three policies; this would also lead to an increase in imports. In addition to increasing domestic consumption, exports would also increase due to increase in production output. Each sector witnesses a different elasticity of imports and exports; the economic impact of imports and exports is also not the same in various sectors. Imports are greater than exports in some industries, whereas exports are stronger than imports in other industries. Meanwhile, the impact of the same policy is not the same on import or export of different sectors. Following the implementation of policies, the imports of finance, electricity, transportation, and fossil fuels increased tremendously; however, the imports of agriculture and heavy industry were not affected significantly. These policies have a greater impact on finance, electricity, construction, and fossil energy exports; however, the same policies have a lesser impact on the exports of heavy industry and agriculture. This is due to the relative fact that China heavily imports following products and services: fossil fuels, oil, and sophisticated transportation equipment, so these sectors have a greater impact. Construction industry always faced the pressure of “destocking,” so there is a phenomenon of not increasing but decreasing in import. China’s trade surplus would be further reduced with the implementation of these three population policies; however, it may also bring about trade deficits.

### The impact of income

Compared to baseline scenario, the three demographic policies have a positive impact on China’s economy (Table 7). The implementation of Policy I can increase household income, corporate income, and government income by 31.0‰, 14.0‰ and 15.2‰, respectively. The implementation of Policy II can rise household income, corporate income, and government income by 34.2‰, 15.4‰ and 16.7‰, respectively. Policy III exerts slight economic impact on these sectors in the short term.

**Table 7:** Income influence.

Policy	Income	2012	2017	2022	2027	2032	2037
Policy Ⅰ	Corporate Income	0.0140	0.0186	0.0163	0.0268	0.0296	0.0250
	Household Income	0.0310	0.0418	0.0370	0.0620	0.0695	0.0592
	Government Income	0.0152	0.0202	0.0177	0.0292	0.0322	0.0272
Policy Ⅱ	Corporate Income	0.0154	0.0247	0.0273	0.0231	0.0224	0.0246
	Household Income	0.0342	0.0557	0.0622	0.0532	0.0524	0.0582
	Government Income	0.0168	0.0269	0.0297	0.0251	0.0243	0.0268
Policy Ⅲ	Corporate Income	0.0000	0.0000	0.0000	0.0000	0.0381	0.0881
	Household Income	0.0000	0.0000	0.0000	0.0000	0.0894	0.2107
	Government Income	0.0000	0.0000	0.0000	0.0000	0.0414	0.0961

However, Policy III exerted a significant economic impact on household income, corporate income, and government income after the year 2032 and the impact is much stronger than Policy I and Policy II. Policy III increases household income, corporate income, and government income by 89.4‰, 38.1‰, and 41.4‰, respectively. Policy I increases household income, corporate income, and government income by 69.5‰, 29.6‰, and 32.2‰, respectively. Policy II increases household income, corporate income, and government income by 52.4‰, 22.4‰, and 24.3‰, respectively.

Compared to baseline scenario, the above result concludes that same policies have a different degree of influence on household income, corporate income, and government income. Moreover, the impact on household income would be stronger than that on government income; the impact on government income would be stronger than that on corporate income. Therefore, we recommend that Chinese government must implement fiscal and taxation measures to increase household, corporate, and government income; the government must also promote second-child policy to increase the birth rate of second child.

## Conclusions and policy implications

In this study, CGE model was constructed to solve China’s aging problem; the main objective of this study was to analyze the economic impact of second-child policy and delayed retirement policy on male or female workers. Our study findings suggest that in the short term, delaying the retirement age imposes a greater impact on the economy. In the long term, second-child policy would effectively tackle the issue of labor shortage; these measures would tremendously improve China’s economy. This helps in optimizing China’s population structure.

All the three policies have a tremendous positive impact on industrial outputs. Agriculture, light industry, finance, and service industry have benefited the most in terms of industrial output; however, heavy industry and construction have been least affected by these policies. In fact, these policies have played a significant role in optimizing and upgrading the industrial structure in China. The three policies have positively impacted industrial imports; however, the construction industry is an exception. These policies would significantly improve following industries: finance, electric power, transportation, and fossil energy. These three policies have tremendously impacted industrial exports; however, agriculture and construction industries are exceptions. Finance, electric power, construction, and fossil energy are the industries that are significantly affected by these three policies. Following the implementation of these policies, imports are more significantly impacted than the exports of all industries; however, agriculture, electric power, construction, and finance are exceptions. The impact of these three policies on household income would be stronger than that on government income. The impact on government income would be stronger than that on corporate income.

According to the analysis of numerical simulations, our policy recommendations are as follows. (1) The three policies do not have any conflict of interest. China’s aging population has a negative impact on the economy. To tackle this problem, retirement age or second-child policy must be implemented effectively. To tackle the problem of insufficient labor supply immediately, the retirement age of both male and female workers give an access to this. By implementing second-child policy, China can effectively solve the problem of labor shortage in the long run; the skewed ratio of demographics can be corrected successfully with the implementation of two-child policy. (2) In different time periods, the impact of delaying retirement age would be different on Chinese economy. The impact of the retirement policy would improve the economy in the short-term; however, the same policy may decline the economy over a period of time. Moreover, the Chinese economy would stabilize over a period of time. To completely solve the problem of labor shortage, Chinese government would have to actively consider implementing second-child policy. (3) Economic fluctuations would be greater if the government only considers delaying the retirement age of either males or females. Therefore, we suggest that the Chinese government should delay the retirement age of both males and females. China's economy can be effectively boosted with this strategy. (4) Regardless of raising the retirement age or implementing the second-child policy, the demand for output and intermediate inputs for agriculture, light industry, finance, and service sectors increases significantly than that of heavy industry and construction. Therefore, it is recommended that when China is implementing these two policies, it should also proactively support the optimization and upgrading of the industrial structure which is the result of implementing the two policies. (5) The two policies would further help in reducing trade surplus; the increase in imports of the same sector would be greater than that of exports. Therefore, China should devise innovative solutions to solve the current trade situation and to facilitate interests in international trade. (6) In the long run, second child policy would be effective in tackling labor shortage; the economy would receive a major boost and national income would increase tremendously. This implies that the Chinese government can adopt corresponding financial and taxation policies to promote the second-child policy.

Some limitations still need to be addressed in future research. First, this study used the static CGE model to evaluate the impact of three population policies on China's economy. In the static CGE model, only the parameter of labor force is dynamically changing, and all other parameters (such as labor capital replacement rate and technical productivity) are values of the base years. This leads to a certain difference between the simulated environment and the actual situation. In the future, we plan to apply the dynamic CGE model to simulate the economic effects of the three policy populations. Second, when using the OLG model to calculate the future labor rate, the birth rate and death rate used in this paper are taken from the base years. In the future, we plan to find out the factors that influence the birth rate and death rate, and then fit the birth rate and death rate to make them more realistic.
